# Simplified Model for the Behaviour of Asphalt Mixtures Depending on the Time and the Frequency Domain

**DOI:** 10.3390/ma18020466

**Published:** 2025-01-20

**Authors:** Péter Primusz, Csaba Tóth

**Affiliations:** 1 Faculty of Forestry, Institute of Geomatics and Civil Engineering, University of Sopron, Bajcsy-Zsilinszky Street 4, H-9400 Sopron, Hungary; 2 Department of Highway and Railway Engineering, Budapest University of Technology and Economics, Műegyetem Rakpart 3, H-1111 Budapest, Hungary; toth.csaba@emk.bme.hu

**Keywords:** master curve, time-frequency equivalency, interconversion, stiffness

## Abstract

Sigmoid functions are widely used for the description of viscoelastic material properties of asphalt mixtures. Unfortunately, there are still no known closed functions for describing connections among model parameters in the time and the frequency domains. In most cases, complicated interconversion methods are applied for the conversion of viscoelastic material properties. To solve this problem, an empirical material model with four parameters has been developed. Parameters of the model can be quickly determined in the frequency domain and can be used in an unchanged way for the description of the material behaviour of the asphalt mixture in the time domain. The new model starts from the mathematical formula of the Ramberg–Osgood material model (short form RAMBO) and its main advantage is that its parameters are totally independent. Model calculations have been performed for the determination of factors necessary for the interconversion in the time and the frequency domains, applying the approximate procedure of Ninomiya and Ferry. The analysis of data has indicated that the interconversion factors in the time and the frequency domains depend only on the slope of the new empirical model function. Consequently, there is no need for further calculations, since the RAMBO model parameters determined in the frequency domain provide an excellent characterisation of the analysed mixture in the time domain as well. The developed new empirical material model has been verified using laboratory data and exact numerical calculations.

## 1. Introduction

The mechanical behaviour of asphalt mixtures for pavement structure design or assessment of in-situ load bearing capacity measurements nowadays is modelled mainly applying the linear viscoelastic theory [1,2,3,4]. Therefore, the main motivation of this scientific work is to provide a tool for engineers dealing with road pavement structures, for simply giving material characteristics both in the time and the frequency domains, required for different analyses and calculations regarding road pavement structures. The asphalt mixture is characterised in the frequency domain by the dynamic modulus and the phase angle, while in the time domain by the creep compliance and the relaxation modulus. The dynamic modulus describes well the behaviour of viscoelastic materials in case of periodic excitation, while the relaxation modulus is more suitable for the analysis of permanent loading [5].

Laboratory determination of material properties in question for a given mixture is a very time-consuming process. A problem may be caused by existing various testing settings for determining the same characteristic [6,7]. Fortunately, the required material characteristics can be well interconverted, since these are linear viscoelastic material properties [8,9]. In the current practice, the dynamic modulus and the phase angle are determined in the laboratory, then the relaxation modulus and the creep compliance are deduced from these by calculation [10]. Therefore, the asphalt stiffness is measured by one type of modulus, while the other types are calculated from this. Interconversion procedures are well prepared; there are a lot of papers concerning this topic [11,12,13], but these procedures require high-level expertise and attentiveness; carrying out them is not a trivial task for most pavement structure engineers.

The aim of this scientific work is to prepare an empirical material model, whose parameters can be determined quickly in the frequency domain, using the Asphalt Mix Performance Tester (AMPT) device manufactured by Cooper Co., Ripley, UK. These parameters are then used in an unchanged way in the time domain for the description of the material behaviour of the asphalt mixture. This possibility is given by the fact that sigmoid functions are suitable for modelling $\left|E^{*}\right|,\;{\;E}^{\prime },\;E^{\prime \prime },\;E\left(t\right),$ and D(t) in practice, although unfortunately closed functions for describing relationships among model parameters are still unknown. The model proposed in this paper provides these function relations, thus enabling their practical use for the design of the pavement structure.

In the process of preparing the new model, firstly a logistic function has been looked for, whose parameters are totally independent from each other [14], substituting well the four parameters master curve model of the dynamic modulus, introduced by [15], very often cited in the literature [16]. Secondly, the question of a direct interconversion between the time and the frequency domains had to be studied. This problem has been analysed earlier by several researchers, but evidently there has been no agreement regarding the time-frequency equivalency factor [5,17,18,19,20,21]. According to the assumption in the presented work, the direct time-frequency interconversion can be executed using only the already determined parameters of the new empirical material model; therefore, there is no need for further surplus calculations.

The presented new approach has been verified using laboratory data and exact numerical calculations.

## 2. Theoretical Background

There are two main categories of master curve models; one is the theoretical (mechanistic) model and the other is the empirical (mathematical) model [22].

Mechanical models use series or parallel coupled systems of springs and dashpots (rheological elements) to create a viscoelastic material reaction. In addition to the generalized Maxwell and generalized Kelvin–Voigt models [23], this includes the popular Huet-Sayegh [24] and its improved S2P1D model [25,26]. The most widely used of the empirical models is the sigmoidal (standard logistic) and its generalised version logistic sigmoidal model to describe the stiffness of asphalt [27]. The physical interpretation of the parameters of empirical models is not always clear, but it is easy to fit them to the data. However, some parameters can be related to the asphalt mix component properties by statistical analysis [15,28]. The type of model presented in this paper is empirical too.

### 2.1. Master Curve for the Stiffness

Master curves of asphalt mixtures are modelled traditionally by sigmoid functions, although other function types could be applied for this purpose as well. According to the recommendation of [14], one such possibility is the Ramberg–Osgood elastic-plastic material law, often used for modelling the cyclic behaviour of soils [29]. The model describes the non-linear connection between the stress and strain of the material about its yield limit value:

(1) $$\varepsilon =\frac{\sigma }{E}+C{\left(\frac{\sigma }{E}\right)}^{R}$$

where ε is specific strain, σ is stress, E is Young’s modulus, C and R are constants depending on the analysed material. The Ramberg–Osgood model is also used to describe the dynamic, non-linear behaviour of soils, because it is suitable for predicting cyclic shear stress-shear strain behaviour [30]. The general mathematical formula of the Ramberg–Osgood model:

(2) $$x=y+Cy^{R}$$

The Ramberg–Osgood model Equation (2) describes magnitude and shape separately by a different model coefficient. The paper of [14] recommends the application of the function (2) for describing the master curve of asphalt mixtures with replacements of $x=f_{r}$ and $y=E_{N}f_{r}$ in the following form:

(3) $$f_{r}=E_{N}f_{r}+C{\left(E_{N}f_{r}\right)}^{R}$$

where $f_{r}$ is the reduced frequency and $E_{N}$ is the normalised dynamic modulus of the asphalt:

(4) $$E_{N}=\frac{E^{*}-E_{e}^{*}}{E_{g}^{*}-E_{e}^{*}}$$

where $E_{e}^{*}$ is the asymptote of low frequencies or high temperatures and $E_{g}^{*}$ is the asymptote of high frequencies or low temperatures. In Kweon’s study, Equation (3) is named as the modified Ramberg–Osgood model of the asphalt mixture master curve. In the rest of this paper, this model type will be abbreviated as RAMBO.

Kweon’s work [14], has proven (p. 38, Table 2) that the parameters of the RAMBO model are really independent from each other opposite to the generally used [15] sigmoid function. This fact has been strengthened by the work of [16], where it is graphically (p. 10, Figure 2) visible that the shape of the master curve is how exactly influenced by sigmoidal parameters. The problem of the usual sigmoid function in practice is that all four parameters influence the inflexion point of the curve.

On the contrary, in the RAMBO model, the R parameter affects only the slope or the curvature of the master curve, while the C parameter—similarly to the temperature-time shift factor—moves the curve along the horizontal axis. This latter statement has not been analysed in detail by the author. Formula (3) reported in the original [14] paper is not sensible for the practice, because it makes the experimental data fitting more difficult. For this reason, we propose the following substitutions $x=f_{r}/E_{N}$ and $y=f_{r}$ in function (2) and it is reduced to achieve $E_{N}$ for further calculations:

(5) $$E_{N}=\frac{1}{Cf_{r}^{R-1}+1}$$

Substituting Equation (4) for $E_{N}$, the final formula for the dynamic modulus is ready:

(6) $$\left|E^{*}\left(f_{r}\right)\right|=E_{e}^{*}+\frac{E_{g}^{*}-E_{e}^{*}}{1+Cf_{r}^{R-1}}$$

**Figure 1:**
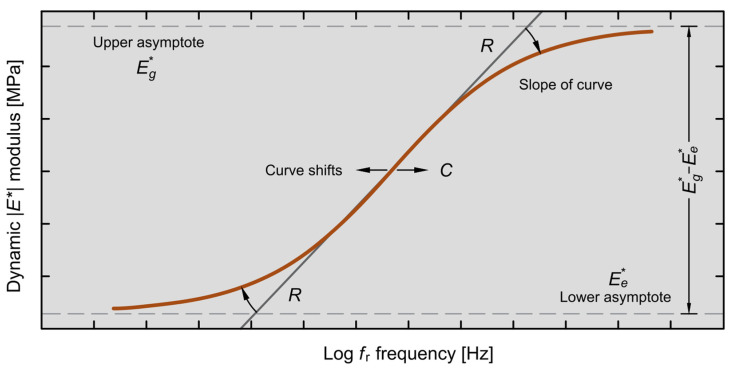
Graph of the dynamic modulus according to Equation (6), indicating parameters.

In Equation (6), the coefficients are separated from each other, so this makes the separation of the asphalt mixture’s dynamic modulus effects. Figure 1 shows graphically the effect of model parameters. The independence of parameters in the RAMBO model makes possible the numerical characterisation of the total morphology of the master curve, providing an opportunity for a quick comparison of various mixture types.

### 2.2. Master Curve for the Phase Angle

The description of the phase angle can be started from the approximate formulae of Kramers–Kronig-relations, introduced by [31]. Later, the original formula was amended by [32], including a c coefficient in order to achieve a potentially better estimation:

(7) $$\phi \left(f_{r}\right)\approx c\frac{\pi }{2}\frac{d\ln\left|E^{*}\left(f_{r}\right)\right|}{d\ln f_{r}}$$

Equation (7) makes it possible to deduce the mathematical model of the phase angle from the master curve of the dynamic modulus [33]. The K-K-relations result in smooth and continuous master curves from the mathematical point of view, but the usage of approximate formulae has certain constraints [34].

Substituting the dynamic modulus formula of the RAMBO model Equation (6) into the approximate formula Equation (7), the sought function of the master curve of the phase angle is given:

(8) $$\phi \left(f_{r}\right)\approx -c\frac{\pi }{2}\frac{\left(E_{g}^{*}-E_{e}^{*}\right)\left(R-1\right)Cf_{r}^{R-1}}{\left(Cf_{r}^{R-1}+1\right)\left(E_{e}^{*}Cf_{r}^{R-1}+E_{g}^{*}\right)}$$

In the case of fitting experimental data including measurement errors into this equation, it is purposeful to complete it by adding further empirical terms, in order to achieve better fitting [35,36]:

(9) $$\phi \left(f_{r}\right)\approx a+b\log\left(f_{r}\right)-c\frac{\pi }{2}\frac{\left(E_{g}^{*}-E_{e}^{*}\right)\left(R-1\right)Cf_{r}^{R-1}}{\left(Cf_{r}^{R-1}+1\right)\left(E_{e}^{*}Cf_{r}^{R-1}+E_{g}^{*}\right)}$$

where a, b, and c are parameters fitted to experimental values. The unlisted parameters are the same as before. In this research, Equation (8) was used to construct the phase angle master curve.

### 2.3. Master Curve for the Relaxation Modulus

The time-dependent relaxation modulus of asphalt mixtures can be empirically approximated by sigmoid shape functions as well, similarly to the dynamic modulus. Consequently, the RAMBO model can be applied for this purpose as well, after some suitable transformation. The relaxation function has in essence the same shape like in Equation (6), having new parameters.

In the literature, there are several procedures and approximate methods for the direct interconversion of viscoelastic material properties from the frequency domain to the time domain [20]. Presently there are two widely used methods in research works for the calculation of the loading time. The first method directly changes the frequency to the loading time using the t=1/f formula. The second method starts with transforming the frequency to the ω angular frequency, then deducts the t=1/2πf loading time. In the case of a general connection between the time and the frequency, only the reverse proportionality of the two variables shall be considered [37]:

(10) $$t=\frac{1}{k_{r}f}=\frac{\beta }{f}$$

where $k_{r}$ is an arbitrary constant and $\beta =1/k_{r}$. Equation (10) shows a general relationship in the case of $k_{r}=1$ constant, which gives the t=1/f and in the case of $k_{r}=2\pi$ gives the t=1/2πf time-frequency interconversion as results. By now the time-dependent relaxation modulus of an asphalt mixture can be described in a general form:

(11) $$E\left(t_{r}\right)=E_{e}^{*}+\frac{E_{g}^{*}-E_{e}^{*}}{1+C{\left(k_{r}t_{r}\right)}^{1-R}}$$

where $E\left(t_{r}\right)$ is the time-dependent relaxation modulus at the $t_{r}$ reduced time point. In order to deduct the time-dependent relaxation modulus from the RAMBO model of the frequency-dependent dynamic modulus, the value of the $k_{r}$ constant must be determined.

### 2.4. Master Curve for the Creep Compliance

For the creep compliance function, the approximate formula modified by [13] has been applied:

(12) $$D\left(t\right)E\left(t^{*}\right)=1$$

where $t^{*}$ is the equivalent time. Park & Kim introduced an α rescaling factor, in order to achieve a more accurate approximate formula, transforming the physical time to the so-called equivalent time [13]. The α rescaling factor can be interpreted as a shift factor on a logarithmic time axis:

(13) $$t^{*}=\alpha t$$

Interconversion relationships for the relaxation modulus and the creep compliance can be exactly described using the equivalent time:

(14) $$D\left(t\right)=\frac{1}{E\left(\alpha t\right)}$$

Substituting the Equation (11) formula for $E\left(t\right)$ and introducing the notation $k_{c}=\alpha k_{r}$ the final model of the creep compliance is as follows:

(15) $$D\left(t_{r}\right)=\frac{1+C{\left(k_{c}t_{r}\right)}^{1-R}}{E_{g}^{*}+E_{e}^{*}C{\left(k_{c}t_{r}\right)}^{1-R}}$$

To construct the master curve of the creep compliance, the value of the $k_{c}$ constant in the formula must be determined.

## 3. Materials and Methods

### 3.1. Presentation of Experimental Data

The RAMBO material model has been tested on four types of asphalt mixtures. The rubber modified (RmB), the polymer modified (PmB), and the reclaimed asphalt contained (RA) mixtures have been analysed as well as a reference mixture with conventional binder (NB):

- AC22 binder NB 50/70 (AG)
- AC22 binder RA 70/100 (FG)
- AC22 binder PmB 25/55-65 (BG)
- AC22 binder RmB 45/80-55 (CG)

The analysed specimens have been mixed using the average value of the lower and upper limits of the AC 22 particle size distribution. The bitumen content was in all cases 4.5 mass % (m%). Dynamic moduli and phase angles of the four different mixtures have been fixed at 20 °C reference temperature applying the Asphalt Mix Performance Tester (AMPT) device. Cyclic tests with cylindrical specimens are not the most accurate method of rheological evaluation when it comes to asphalt mixes, due to the uniaxial loading application mode. Nevertheless, they remain one of the most prevalent and accepted test methods for the design of road pavement structures for asphalt mixtures. This is precisely why the primary focus of our efforts has been on this test. Three laboratory specimens have been prepared from each mixture for further detailed testing and their average values have been used. A comprehensive overview of the asphalt mixtures, their preparation, and the test procedure can be found in our previous work on [38].

### 3.2. Factors of Interconversion in the Time and the Frequency Domains

Model calculations have been performed to determine the $k_{r}$ and $k_{c}$ constants necessary for the time-frequency interconversion. A relationship has been looked for between parameters of the RAMBO model and the $k_{r}$ and $k_{c}$ constants in question, using an empirical method. The main steps of the method applied in this work are the same as described in the study of [39].

As a first step, the parameters of the RAMBO model of the dynamic modulus have been varied in a wide range, establishing a hypothetic $E^{*}\left(f_{r}\right)$ master curve database. All possible combinations of parameters in Table 1 have been applied in the analysis, finally putting together a test database of totally 400 master curves. The next step consisted of the determination of the relaxation modulus and the creep compliance for all $E^{*}\left(f_{r}\right)$ and $\phi \left(f_{r}\right)$ master curves, applying approximate formulae of Ninomiya & Ferry [40]:

(16) $$E\left(t\right)\approx E^{\prime }\left(\omega \right)-0.40E^{\prime \prime }\left(0.40\omega \right)+0.014E^{\prime \prime }\left(10\omega \right){|}_{t=1/\omega }$$

(17) $$D\left(t\right)\approx D^{\prime }\left(\omega \right)+0.40D^{\prime \prime }\left(0.40\omega \right)-0.014D^{\prime \prime }\left(10\omega \right){|}_{t=1/\omega }$$

Park & Schapery deduct the approximate formulae for the relaxation modulus likewise from dynamic data [41]. Calculations of Abdollahi et al. have shown that formulae of the two approximate procedures provide nearly equal results, so the simpler one has been chosen [42].

Every master curve has 50 data points within the frequency (or time) domain of 10^−7^ and 10^+7^. For all $E\left(t_{r}\right)$ master curves in the database, Equation (11) has been fitted, where only the $k_{r}$ constant remained unknown.

For all $D\left(t_{r}\right)$ master curves in the database, Equation (15) has been fitted, where only the $k_{c}$ constant remained unknown. The goodness of fit has been measured by the Root Mean Square Error, RMSE:

(18) $$RMSE=\sqrt{\frac{1}{N}\sum_{i=1}^{N}{\left(Y_{i}-\hat{Y_{i}}\right)}^{2}}$$

A regression analysis has been performed to determine a relationship of $k_{r}$ and $k_{c}$ constants with R and C parameters of the RAMBO model. Finally, equations described for $k_{r}$ and $k_{c}$ constants have been simplified on the best way while retaining their accuracy.

### 3.3. Applicability Analysis of the RAMBO Model

Applicability analysis of the freshly developed RAMBO model has been performed both by comparative calculations and real test results. The main aim of the validation has been to assess accurately the prediction ability of the RAMBO model. As first step, viscoelastic comparative calculations has been performed using the ELiCon v0.1 Microsoft Excel worksheet [43], suitable for real-time exact interconversion between the time and the frequency domains. The input of the ELiCon v0.1 in the time domain is a four-parameter analytic function, recommended by [44], to describe the creep compliance:

(19) $$D\left(t\right)=D_{\infty }+\frac{D_{0}-D_{\infty }}{1+{\left(t/\tau_{D}\right)}^{n_{D}}}$$

where $D_{0}\;$ determines the upper asymptote of the curve, $D_{\infty }$ determines the lower asymptote of the curve, $\tau_{D}$ shifts horizontally the total curve to the left or right, and $n_{D}$ determines the slope of the curve between the lower and upper asymptotes. Equation (19) above is very similar to Equation (6) and is therefore a RAMBO type model.

The ELiCon v0.1 worksheet applies Equation (19) for the calculation of the uniaxial creep compliance for given points of time; moreover, it provides the relaxation modulus values for the same points of time. In the frequency domain, the outputs of the worksheet for certain frequencies are the absolute value of the complex modulus (the dynamic modulus) and the phase angle. The interconversion is based on the Prony-series of the generalised Maxwell model [41,45]. Master curves of the dynamic modulus and the phase angle in the frequency domain, as well as master curves of the relaxation modulus and the creep compliance in the time domain, have been calculated using the ELiCon v0.1 worksheet with parameters given in Table 2. After this, the RAMBO material model has been fitted by the Equation (15) relationship to the $D\left(t\right)$ master curve of the creep compliance given by the ELiCon v0.1 worksheet and predicted the other three material properties functions using the RAMBO parameters ($E_{e}^{*}$, $E_{g}^{*}$, R, and C), determined by the fitting procedure. This procedure has been performed for the master curves of the relaxation modulus, the dynamic modulus, and the phase angle as well. In all four master curve groups 4 × 4 × 4 = 64 RAMBO models have been established and compared to the results of the ELiCon v0.1 worksheet.

In the second step after synthetic data, the RAMBO model has been fitted to real test results. For the temperature shift function the log-linear model has been chosen:

(20) $$\log\left(\alpha_{T}\right)=B\left(T-T_{0}\right)$$

where B is the slope of the linear line between $\log\left(\alpha_{T}\right)$ and the temperature, $T_{0}$ is the reference temperature. Since the $E^{*}\left(f_{r}\right)$ dynamic modulus and the $\phi \left(f_{r}\right)$ phase angle are strictly related to each other, the Equation (6) dynamic modulus and the Equation (8) phase angle formulae of the RAMBO model have been fitted simultaneously to the test data, minimising the Root Mean Squared Relative Error, RMSRE:

(21) $$RMSRE=\frac{1}{N}\sqrt{\sum_{i=1}^{N}{\left(\frac{E_{m,i}^{*}-E_{p,i}^{*}}{E_{m,i}^{*}}\right)}^{2}}+\frac{1}{N}\sqrt{\sum_{i=1}^{N}{\left(\frac{\phi_{m,i}-\phi_{p,i}}{\phi_{m,i}}\right)}^{2}}$$

where $E_{m,i}^{*}$ and $\phi_{m,i}$ are the dynamic modulus and the phase angle measured at the i. test, $E_{p,i}^{*}$ and $\phi_{p,i}$ are the i. dynamic modulus and phase angle predicted by the RAMBO model, and N is the number of tested specimens. Relative error functions have been used because the dynamic modulus and the phase angle differs from each other by several magnitudes. The optimisation has been performed using the Solver v2.7 extension of Microsoft Excel. Out of model parameters, it is practical to give initial values of the $E_{min}^{*}$ and the $E_{max}^{*}$ as the minimum and maximum dynamic modulus values of test results, if there is no other rational constraint. The results of the model fitting process are in this case the $E_{e}^{*}$, $E_{g}^{*}$, R, and C parameters of the RAMBO model as well.

### 3.4. Assessment of the Quality of the Model Fitting

In the case of all regression models, the standard error ($S_{e})\;$ of the estimation of Y relevant to X, the standard deviation ($S_{y}$) and the adjusted coefficient of determination ($R_{*}^{2}$) has been calculated. The standard error of estimation has been calculated as follows:

(22) $$S_{e}=\sqrt{\frac{\sum_{i=1}^{N}{\left(Y_{i}-{\hat{Y}}_{i}\right)}^{2}}{N-M-1}}$$

where N is sample size, M is number of independent variables, fitted parameters, $Y_{i}$ is measured value, and ${\hat{Y}}_{i}$ is predicted value. The standard deviation of the sample is:

(23) $$S_{y}=\sqrt{\frac{\sum_{i=1}^{N}{\left(Y_{i}-{\bar{Y}}_{i}\right)}^{2}}{N-1}}$$

where ${\bar{Y}}_{i}$ is the average of measured values; the others are as above. The $S_{e}/S_{y}$ standard error ratio and the adjusted coefficient of determination ($R_{*}^{2}$) have been used for the assessment of the quality of fit between measured and predicted values. The adjusted $R_{*}^{2}$ is a modification of the $R^{2}$, taking into account the number of observations and the number of explanatory variables:

(24) $$R_{*}^{2}=1-{\left(\frac{S_{e}}{S_{y}}\right)}^{2}$$

The smaller is the $S_{e}/S_{y}$ and the higher is the $R_{*}^{2}$, the better is the fit of predicted and measured data. In the case of model construction, maximising $R_{*}^{2}$ is equal to minimising the residual variance; therefore, that model is worth choosing, where $R_{*}^{2}$ is maximal. Based on former research, the model fit is excellent in the case of the value of the $S_{e}/S_{y}$ ratio is less than 0.35, and the value of the $R_{*}^{2}$ is more than 0.9, according to Table 3 [46]. Finally, it is worth to consider that a high $R_{*}^{2}$ does not mean that the model is good, although its low value indicates at a high probability that the model is wrong.

## 4. Evaluation of Results

**Figure 2:**
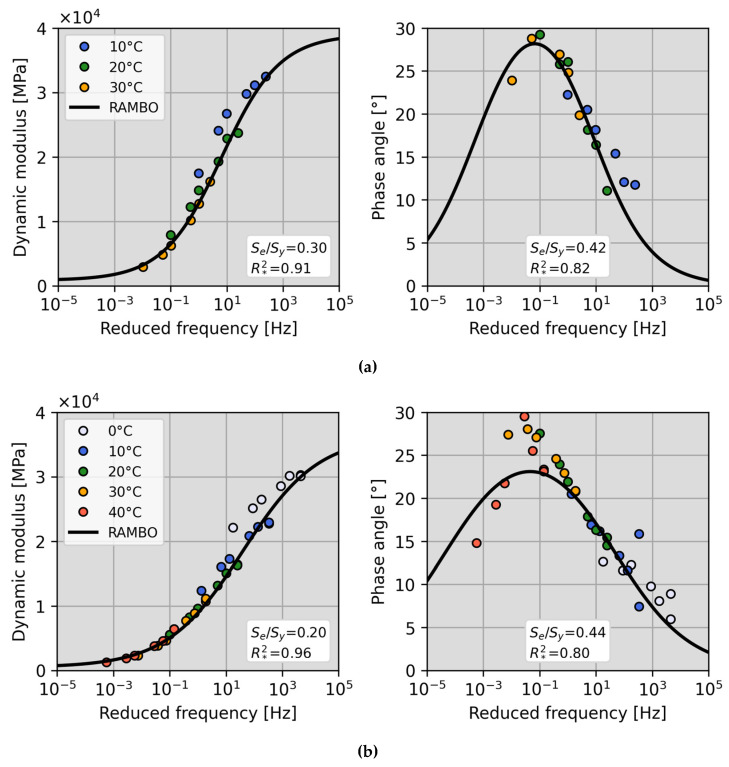
Average master curves of the dynamic modulus and the phase angle at $T_{0}=20\;{}^{\circ}C.$ The asphalt mixtures tested: (**a**) AG–NB 50/70 and (**b**) GG–RA 70/100.

The RAMBO model has been fitted to real test data to prove its ability to describe master curves of asphalt mixtures known from the literature. Figure 2 shows graphically an arbitrary example of the fitting of the master curves of the dynamic modulus and the phase angle in case of analysed test mixtures. Figure 2b illustrates the scattered correlation between the phase angle and the reduced frequency, highlighting the dominant viscous behaviour of the asphalt mixture. This pattern can be attributed to the limitations of the RAMBO model in capturing the reduced stiffness of asphalt mixtures under conditions where the viscous component prevails, particularly at elevated temperatures and low frequencies.

Table 4 contains average parameters of the RAMBO model for each mixture. The model parameters are employed principally for the purpose of demonstration. It is important to note that they are not intended for general use; rather, their primary function is to illustrate the validity of the RAMBO model in describing experimental data.

It can be stated in general that most master curves of the $\left|E^{*}\right|$ dynamic modulus predicted by the RAMBO model happened to be within the excellent fit category. The RAMBO model of the phase angle has somewhat poorer performance, but the goodness of fit is still acceptable, based on Table 3. The arbitrary chosen log-linear $\alpha_{T}$ shift factor for describing master curves has performed well for all analysed asphalt mixtures.

The RAMBO model overall has provided good prediction for dynamic modulus values; still, it slightly fell behind the sigmoid function widespread in practice. The main reason is that in the present study, for the determination of parameters of the RAMBO model, phase angle data have been considered as well, decreasing the predictive ability of the model (while increasing its physical content). Phase angle values predicted by the RAMBO model have shown a strong linear correlation with measured data, although the predicted phase angle values mainly have been slightly below measured ones ($R^{2}=0.896$). Results have shown overall that the RAMBO model still predicts well the phase angle.

To determine factors for the time-frequency interconversion, a multiple linear regression has been performed on data of the hypothetic master curve database:

(25) $$k_{r}=\beta_{0}+\beta_{1}R+\beta_{2}C$$

where $\beta_{0}$ is axis intersection, and $\beta_{1}$ and $\beta_{2}$ are regression parameters. Parameters and statistical features of the model have been determined by Microsoft Excel Data Analysis:

(26) $$k_{r}=16.3349-7.0396R-0.0124C$$

Since the value of the $\beta_{2}$ parameter is practically zero, the value of the $k_{r}$ parameter is only slightly affected by the C parameter; therefore, the final formula can be simplified as follows:

(27) $$k_{r}=16.31-7.04R$$

Steps of analysis shown for the relaxation modulus have been performed for data series of the creep compliance as well. In the case of the creep compliance, the determination of the $k_{c}$ constant is possible by the following simple empirical formula:

(28) $$k_{c}=5.15+6.26R$$

Table 5 and Table 6 summarise statistical characteristics of the simplified (27) and (28) models.

Based on the (27) relationship, it can be stated that the interconversion of viscoelastic material properties of asphalt mixtures from the frequency domain to the time domain depends not only the reverse proportionality of the variables but also from the R parameter of the RAMBO model, which determines the slope of the master curve between the lower and upper asymptotes. The known $k_{r}$ and $k_{c}$ constants provide a possibility for the interconversion of viscoelastic material properties from the frequency domain to the time domain. Table 7 summarises the calculated time-frequency interconversion factors of the RAMBO model.

Furthermore, taking into account the typical values of the R parameter of the analysed asphalt mixtures (0.57–0.67 according to Table 4), it can be stated that the $k_{r}$ factor varies only in a narrow domain (between 11.6 and 12.3); therefore, the loading time may have a good approximation using formulae t≈0.09/f or a t≈0.08/f.

In the literature a work [7] can be found where the relationship between the loading time and the angular frequency [47] has been applied for the interconversion:

(29) $$\omega =\frac{1}{2t}$$

From this formula an approximate formula for the loading time can be deducted substituting ω=2πf:

(30) $$t=\frac{1}{4\pi f}\approx \frac{0.08}{f}$$

A further verification is given by examining data from Table 3 on page 14 in the work of [19]. It is visible that the approach of [18] is nearly in agreement with the approximate value deducted from the RAMBO model. Furthermore, it is worth to mention the approximate relationship deducted by [18], determining the interconversion factor as $1/\pi^{2}\approx 0.1$ similarly. The most recent results in this question can be found in the study of [5], where the optimal interconversion factor for the four parameters sigmoid function introduced by [15] is given as t≈0.0673/f based on the data of 30 asphalt specimens. This interconversion factor is valid for the R≈0.314 parameter of the RAMBO model that is unfortunately out of the analysis limits of the present study. It is presumable that the interconversion factor is not totally independent from the mathematical characteristics of the chosen empirical master curve function.

**Figure 3:**
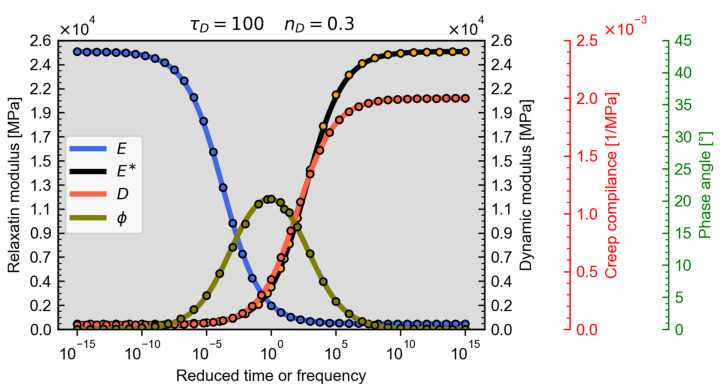
Example for fitting the RAMBO master curves to the results of the ELiCon v0.1 worksheet. The solid curves represent the individual RAMBO model master curves, while the circles represent the ELiCon results.

The calculation method has been compared to the Prony-series calculated by the ELiCon v0.1 worksheet based on the generalised Maxwell-model. The RAMBO material model has been fitted to the calculated data points, Figure 3, showing an arbitrary example of the fit.

**Figure 4:**
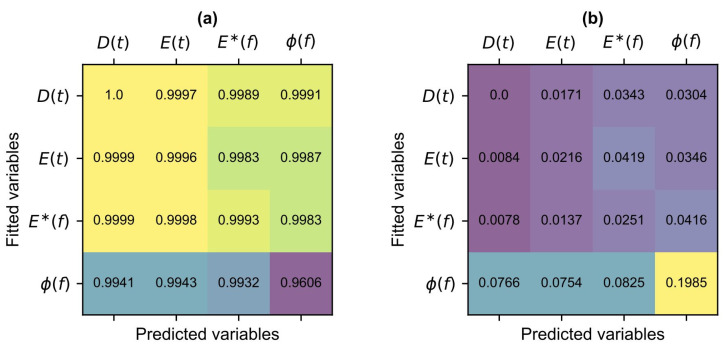
Accuracy matrices of the RAMBO model: (**a**) adjusted coefficient of determination and (**b**) standard error ratio. Note: in the figure, the colours are used to group the results and have no additional significance attributed.

The goodness of fit of the Prony-series calculated by the ELiCon v0.1 worksheet and the empirical RAMBO model has been measured using the adjusted coefficient of determination and the standard error ratio. In the four master curve groups there were 4 × 4 = 16 master curves and the comparative matrices of Figure 4 include the lowest values of the characteristics.

Generally, it can be stated, that all master curve groups remained in the excellent category based on Table 3. In the case of relative comparisons of certain groups, the most “unfavourable” characteristics have been found at phase angle master curves.

This fact can be explained because the Prony-series approximation of the ELiCon v0.1 worksheet is mostly including errors in this case. These numerical errors as well as test errors give a warning that starting only from the phase angle, it is not worth to predict the other viscoelastic material properties. Based on synthetic data, it can be stated that the empirical based RAMBO model can be well used in both the time and the frequency domains, since its parameters are not changing.

## 5. Summary and Conclusions

In the present study a possible new method for the construction of the master curve of the asphalt mixture has been presented, applying the RAMBO model. According to the analysis performed, the RAMBO model can substitute well the sigmoid function for the description of the master curve of the dynamic modulus. It has an important advantage against the sigmoid function since its parameters are independent from each other. Our model has been extended to describe the phase angle as well, applying Kramers–Kronig relations. Comparative calculations show that the new phase angle function fits very accurately to results of the generalised Maxwell-model approximated by Prony-series. Moreover, beyond theoretical calculations, the fit of RAMBO models for the dynamic modulus and the phase angle has been compared to laboratory data as well. While the goodness of fit has fallen behind some very high values found in the literature, it is still can be seen as suitable for the practice. An important remark is that as model parameters have been fitted simultaneously to the data of the dynamic modulus and the phase angle, the lower than usual fitting quality can be explained mainly by the higher uncertainty of phase angle data.

The interconversion of the RAMBO model from the frequency domain to the time domain has been developed. Theoretical calculations have shown that the interconversion of viscoelastic material properties from the frequency domain to the time domain depends on the R model parameter, determining the slope of the master curve between its lower and upper asymptotes. This fact indicates that the time-frequency interconversion factor is not constant since its value depends on the material quality of the asphalt mixture. The $k_{r}\left(R\right)$ function developed for the $E\left(t\right)$ relaxation modulus has been validated using an E(t) derived from an almost accurate Prony-series. A similar approach has been taken in case of the $k_{c}\left(R\right)\;$ function developed for the creep compliance. A total agreement has been found between the master curves of the RAMBO model for the relaxation modulus plus the creep compliance and the Prony-series approximation of the ELiCon v0.1 worksheet. These comparative calculations have shown that the model parameters of the RAMBO model determined in the frequency domain characterise exceedingly the analysed mixture in the time domain as well.

It is acknowledged that the number of asphalt mix types that have been tested and used in the simulations is inadequate for the full validation of the model presented in this article. However, it is crucial to underscore that the primary objective of this study was the development of a novel model that is both reliable and user friendly, specifically for the design of road pavements. The aim was not to create a universally applicable model, but rather to introduce a novel approach to calculations. This study sought to demonstrate the RAMBO model’s ability to accurately describe the experimental data.

However, it should be noted that the present study did not extend to a comparison with laboratory data concerning relaxation modulus and creep compliance. Furthermore, comparative analyses with other types of loading modes and geometries are also planned, including trapezoidal (two-point bending test) and prismatic beams (four-point bending test). This analysis is deemed essential for future research in this field.

In addition, a significant research direction could involve the exploration of the relationships between the parameters of the RAMBO model and the physical properties of the binder in the analysed asphalt mixtures.

## Figures and Tables

**Table 1:** Parameters of the RAMBO model used for establishing master curves of the dynamic modulus and the relaxation modulus.

Parameters	Value
$E_{e}^{*}$ [MPa]	0, 100, 200, 300
$E_{g}^{*}$ [MPa]	25,000, 30,000, 35,000, 40,000
R	0.5, 0.6, 0.7, 0.8, 0.9
C	1.0, 1.5, 2.0, 2.5, 3.0

**Table 2:** Model parameters of the master curves calculated using the ELiCon v0.1 worksheet.

Parameters	Value
$D_{0}$ [1/MPa]	1/25,000
$D_{\infty }$ [1/MPa]	1/500
$\tau_{D}$	10, 10^2^, 10^3^, 10^4^
$n_{D}$	0.2, 0.3, 0.4, 0.5

**Table 3:** Criteria for the assessment of fit.

Criteria	$R_{*}^{2}$	$S_{e}/S_{y}$
Excellent	≥0.90	≤0.35
Good	0.70–0.89	0.36–0.55
Fair	0.40–0.69	0.56–0.75
Poor	0.20–0.39	0.76–0.89
Very Poor	≤0.19	≥0.90

**Table 4:** Characteristics of RAMBO master curves fitted to laboratory test data.

Mixture Type	Code	Model Parameters					Dyn. Modulus		Phase Angle	
		E_min_	E_max_	C	R	B	Adj. R^2^	S_e/_S_y_	Adj. R^2^	S_e/_S_y_
NB 50/70	AG	00831	39 008	2.1920	0.5799	−0.0990	0.91	0.30	0.82	0.42
PmB 25/55–65	BG	1 054	33 646	1.9303	0.5984	−0.0983	0.88	0.35	0.70	0.55
RmB 45/80–55	CG	1 728	21 101	2.8505	0.5194	−0.1042	0.95	0.22	0.70	0.52
RA 70/100	FG	00472	36 343	3.2522	0.6773	−0.1124	0.96	0.20	0.80	0.44

**Table 5:** Statistical characteristics of the $k_{r}$ parameter of the multivariate regression analysis.

	Coefficients	Standard Error	t Stat	*p*-Value	Lower 95%	Upper 95%
Intercept	16.3100793	0.00809372	2015.15192	0	16.2941675	16.3259911
X Variable	−7.0396789	0.01133348	−621.14023	0	−7.0619599	−7.017398

**Table 6:** Statistical characteristics of the $k_{c}$ parameter of the multivariate regression analysis.

	Coefficients	Standard Error	t Stat	*p*-Value	Lower 95%	Upper 95%
Intercept	5.14638265	0.02194529	234.509668	0	5.10323948	5.18952582
X Variable	6.2635073	0.03072955	203.826831	0	6.20309477	6.32391983

**Table 7:** Time-frequency interconversion factors of the RAMBO model.

R	$k_{r}$	$k_{c}$	α	β
0.5	12.81	8.34	0.651	0.078
0.6	12.08	8.90	0.737	0.083
0.7	11.37	9.46	0.832	0.088
0.8	10.67	10.08	0.945	0.094
0.9	9.99	10.88	1.089	0.100

## Data Availability

The original contributions presented in this study are included in the article. Further inquiries can be directed to the corresponding author.
